# The Morphological and Hemodynamic Characteristics of the Intraoperative Ruptured Aneurysm

**DOI:** 10.3389/fnins.2019.00233

**Published:** 2019-03-26

**Authors:** Qingyuan Liu, Pengjun Jiang, Jun Wu, Bin Gao, Shuo Wang

**Affiliations:** ^1^Department of Neurosurgery, Beijing Tiantan Hospital, Capital Medical University, Beijing, China; ^2^China National Clinical Research Center for Neurological Diseases, Beijing, China; ^3^Center of Stroke, Beijing Institute for Brain Disorders, Beijing, China; ^4^Beijing Key Laboratory of Translational Medicine for Cerebrovascular Diseases, Beijing, China; ^5^School of Life Sciences and Bioengineering, Beijing University of Technology, Beijing, China

**Keywords:** intraoperative aneurysm rupture, hemodynamics, morphology, computational fluid dynamics, aneurysm clipping

## Abstract

**Background and Objectives:**

Intraoperative aneurysm rupture (IOR) is a difficult event during the clipping process for intracranial aneurysms, and could result in a bad prognosis. Preoperative discrimination of aneurysms with high risk of IOR is vital for operators. The aim of this study was to explore the hemodynamic-morphological risk factors for the IOR.

**Methods:**

In the present study, patients admitted for unruptured IA from January 2012 to April 2018 were retrospectively reviewed. A propensity score matching was performed to match patients. The morphological features and the hemodynamic features were extracted. Differences in the morphologic and hemodynamic parameters were compared. Risk factors associated with IOR were explored. Subsequently, the hemodynamic characteristics in different rupture stages and different regions in IOR aneurysm were compared.

**Results:**

96 cases of patients with aneurysms, were found by the matching process in each group. The statistically significant difference was found in the maximum length (L) (*p* = 0.041), maximum diameter of body (D) (*p* = 0.032), aspect ratio (AR) (*p* < 0.001), non-sphericity index (NSI) (*p* < 0.001), normalized wall shear stress maximum (NWSSm) (*p* < 0.001) and oscillatory shear index (OSI) (*p* < 0.001). A regression analysis demonstrated AR (OR = 7.03, *p* < 0.001), NWSSm (OR = 15.55, p = 0.014) and OSI (OR = 28.30, *p* < 0.001) as the independent risk factors for IOR. AR was much larger, and NWSSm and NWSSa were much lower for IAs that ruptured in early or pre-dissection stage than those for IAs that ruptured in dissection stage or clip application stage. NWSSa and NWSSm in rupture area were both lower than those in adjacent area.

**Conclusion:**

AR, NWSSm, and OSI are considered three independent risk factors for intraoperative aneurysm rupture, which could serve as predictors. A selection of intervention methods for aneurysms with high AR, low NWSSm, and high OSI should carefully be considered.

## Introduction

Thus far, microsurgical clipping remains one of the major treatments for intracranial aneurysm (IA), which can effectively prevent an aneurysm from rupturing (Lawton and Vates, 2017; Walendy and Stang, 2017). Intraoperative rupture (IOR) occurs in 2.84–8% of IAs during microsurgical clipping (Farnoush et al., 2013; Lakicevic et al., 2015; Chen et al., 2016). The hemorrhage caused by an aneurysm rupture can quickly “submerge” the surgical region, leading to not only panic and iatrogenic injury but also to a potentially catastrophic outcome (Lakicevic et al., 2015). Even experienced neurosurgeons can hardly avoid the incident of IOR. An IOR could result in a poor outcome. According to a previous study by Samson et al., more than 30% of the patients suffering an intraoperative rupture had a poor outcome. Although great advancements in surgical techniques have been achieved during the past few decades, the occurrence of IOR still results in a high incidence of poor outcomes. A recent study reported a 50% poor outcome rate for patients with an IOR aneurysm, suggesting that the IOR can lead to severe adverse prognosis. Thus, how to identify IAs with a high risk of IOR is of great significance.

The intraoperative rupture occurs in three stages, known as the early or pre-dissection stage, dissection stage and clip application stage (Batjer and Samson, 1986). However, since there is no good method to study this problem, only a few related clinical studies have explored its risk factors. Factors (e.g., surgical experience) may affect the risk of IOR. A presurgical rupture is admittedly a high-risk factor for IOR (Lakicevic et al., 2015). A clinical retrospective study reported that being male and having a history of seizures could rise the risk of IOR (Lakicevic et al., 2015). Several other studies found a relationship between the localization of an aneurysm and IOR. A high IOR rate was found in IAs in anterior communicating artery and middle cerebral artery (Schramm and Cedzich, 1993; Leipzig et al., 2005). However, to interpret this phenomenon, the authors argue that the aneurysms have a more fragile wall besides the problems of the operation, yet no studies have been performed to prove this argument. Thus, the reason for IOR still remains unclear.

Previously, Dhar found a relationship between the AR and the risk of nature rupture of IAs. In a 4-dimensional MR study, Gu found that IAs with a larger AR have a higher risk of IOR. Also, Qiu thought that the IA with larger AR may have a more fragile wall (Qiu et al., 2017). In addition, a recent case study reported that the site of IOR was located in the low WSS area (Yoshiki et al., 2017). Given this, Meng proposed that low WSS could cause apoptosis, endothelial injury and inflammatory infiltration (Meng et al., 2014). Those pathological factors would result in atherosclerosis and a fragile aneurysm wall. It can be seen therefore, that an IOR aneurysm has unique morphological and hemodynamic features.

To investigate the hemodynamic-morphological risk factors for the IOR, a group of patients with unruptured IAs was retrospectively reviewed, and their hemodynamic-morphological features were analyzed.

## Materials and Methods

### Patient Selected and Inclusion/Exclusion Standards

In the present study, the patients admitted into our hospital for unruptured IA between January 2012 and April 2018 were retrospectively reviewed. The criteria for selecting patients were as follows:

Inclusion criteria: (1) only one IA was treated if multiple IAs existed (2) no presurgical rupture history (3) complete clinical records (4) presurgical CTA image data (5) microsurgical clipping was the only treatment.

Exclusion criteria: (1) patients had other intracranial tumors, angiostenosis and angio-malformation, including arteriovenous malformation, cavernous malformation, etc. (2) CTA data was not suitable for morphological analysis or hemodynamic analysis. (3) History of neurosurgical surgery (4) recurrent IA (5) the distance between aneurysms was too close if there existed multiple aneurysms.

### Surgery and Definition

To avoid the effect of experience, all microsurgical clippings of IA were performed by two experienced senior neurosurgeons (SW and YC have both worked as neurosurgeons for more than 20 years).

The definition of IOR with any visible bleeding was used here, which included bleeding that stops and changes the order of microsurgical procedures; trivial leaks and minor bleeding which is easy to control surgically.

### Vascular Modeling

The DICOM data of the last CTA, conducted before surgery, were collected from the high-solution CTA workstation (Siemens, Berlin, German) and converted into slice DICOM data (about 0.5 mm per slice). The DICOM data were introduced into Mimics 17.0 (Mimics Research 17.0, Materialize, Belgium) and reconstructed for further study.

### Radiological Measuring and Morphological Parameter

**FIGURE 1:**
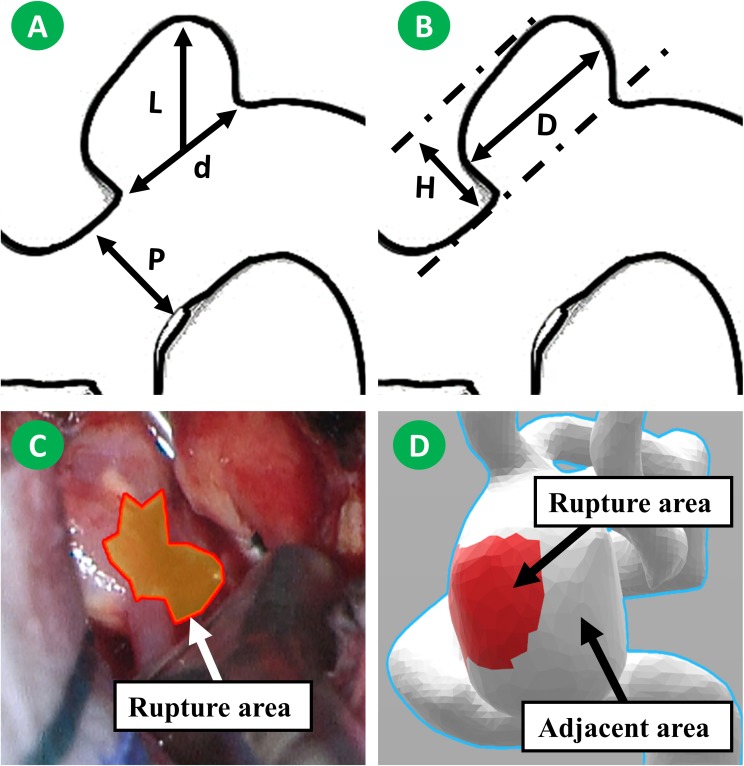
Morphological parameters were measured from CTA image. H is the maximum perpendicular distance of the dome from the neck plane. L is the maximum distance of the dome from the neck plane. d is the average diameter of the neck. p is the average diameter of the parent artery. D is the maximum diameter of the body **(A,B)**. We have roughly matched the rupture area on the vascular model. Subsequently, the hemodynamic changes in rupture area and adjacent area were monitored **(C,D)**.

Radiological measuring was performed by two experienced neurosurgeons (PJ and JW) using a high-solution CTA. L, D, d, H, and aneurysm volume were measured from CTA as shown in Figure 1A,B. (H) is the maximum perpendicular distance of the dome from the neck plane. (l) Represents the maximum distance of the dome from the neck plane. (d) Is the average diameter of the neck. (p) Is the average diameter of the parent artery. (D) Is the maximum diameter of the body. Those parameters were measured twice, and the average taken. AR, size ratio (SR), undulation index (UI), EI, and NSI were calculated according to a previous study (Dhar et al., 2008).

### Computational Fluid Dynamic (CFD) Simulating and Hemodynamic Parameter

To create 4 to 5 million unites of finite tetrahedral and prism elements, each aneurysm model was meshed by STAR-CCM (STAR-CCM+ 12, Siemens, German). Subsequently, the models were introduced into the STAR-CCM fluid workstation (STAR-CCM+ 12, Siemens, German). Based on the previous study (Xiang et al., 2011), the incompressible Navier-Stokers equation served as the solver under a pulsatile blood condition. The pulsatile waveform was obtained with a transcranial Doppler ultrasound device on a representative patient, of which the magnitude was scaled to the desired mean flow rate. This waveform was plotted to the pulsatile curve. The pulsatile waveforms of the internal carotid artery and basilar artery were employed for further analysis. Blood was assumed as a Newtonian fluid with a density of ρ = 1056 kg/m^3^ and viscosity of μ = 0.0035 Poise. The pulsatile curve was taken as the velocity inlet boundary condition, and the free boundary condition was implemented at the outlet. When the residuals were less than 10^−5^, the results were considered converged (Tian et al., 2016). Four pulsatile cycles were simulated. Results from the last cycle were yielded for further study.

For each model, the pressure maximum (Pm), pressure average (Pa), WSS maximum (WSSm), and WSS average (WSSa) was obtained from the IA region, and the parent pressure average (pPa) and parent WSS average (pWSSa) was taken from the parent artery region. The WSS average and pressure average at the peak of the systolic phase were applied for further study. Low WSS was defined as less than 10% of WSS of the parent artery (Xiang et al., 2011). The NPa, NWSSa, NPm, and NWSSm was calculated by the equation (①②③④), respectively. OSI and RRT was calculated according to the previous study. OSI and RRT refer to the average over the dome area.

$$\begin{array}{cc} \mathrm{NPa}=\frac{\mathrm{Pa}}{\mathrm{pPa}}---① & N\mathrm{WSSa}=\frac{\mathrm{WSSa}}{\mathrm{pWSSa}}---②\\ \mathrm{NPm}=\frac{\mathrm{Pm}}{\mathrm{pPm}}---③ & N\mathrm{WSSm}=\frac{\mathrm{WSSm}}{\mathrm{pWSSm}}---④ \end{array}$$

### Mapping Analysis Between the IOR Area and Non-IOR Area

To study the hemodynamic features of the rupture area in depth, the rupture area was roughly matched on the vascular model. Subsequently, the hemodynamic changes in the rupture area and the adjacent area were monitored (Figure 1C,D). The monitoring indicators included NWSSa, NWSSm, and OSI. Next, the differences of those hemodynamic parameters between different areas were compared.

### Propensity Score Matching

To exclude known clinical risk factors and to balance the baseline, a propensity score matching (PSM) was performed to match patients with STATA (12SE, STATA corporation, American). The propensity scores were calculated using a logistic regression model consisting of the input variables: seizure history, gender and localization. The matching rate was 1:1 for IOR IAs to non-IOR IAs. The final matched group was proven to be properly matched using *χ*^2^ testing to validate the equivalence of individual variables between all groups.

### Statistical Analysis

Categorical variables were compared by the chip-square test or Fisher’s exact test. Continuous variables were first assessed visually by the P-P-plots and the Shapiro-Wilk test, and then compared by the independent sample’s *t*-test. A univariate logistic analysis was performed to find the risk factors for the IOR. A multivariate logistic analysis was then performed to demonstrate the independent risk factors. Assuming that a *p*-value of <0.05 is statistically significant. The result was expressed in 95% confidence interval. All statistical analysis was conducted using SPSS 22.0 (IBM, New York, NY, American).

## Results

### Patient Characteristics

Finally, 2237 patients with unruptured IA were reviewed. A total of 96 IORs were identified. The incident rate of IOR was 4.3%. Four IORs occurred in the early or pre-dissection stage, 42 IORs occurred in dissection stage, and 50 IORs occurred on the clip application stage. The PSM yielded 192 patients, including 96 IOR aneurysms and 96 non-IOR aneurysms. A *χ*^2^ testing validated the equivalence of male gender, seizure history and localization between each group.

Comparing patient characteristics, no statistical difference was found. The two groups showed similar age (*p* = 0.554), hypertension history (*p* = 0.409), atherosclerosis history (*p* = 0.371) and smoking history (*p* = 0.382). The demography information is listed in Table 1.

**Table 1:** Demography information of enrolled patients.

	IOR	Non-IOR	*p*
	
Characteristics	*n* = 96	*n* = 96	Value
Gender			N/A
Male	57	57	
Female	39	39	
Mean age (years)	46.3 ± 11.4	44.1 ± 9.8	0.554
Seizure history			N/A
YES	14	14	
NO	82	82	
Hypertension history			0.409
YES	26	19	
NO	70	77	
Atherosclerosis history			0.371
YES	14	26	
NO	82	70	
Ever-or-now smoker			0.382
YES	35	28	
NO	61	68	

### Morphological and Radiological Differences

**FIGURE 2:**
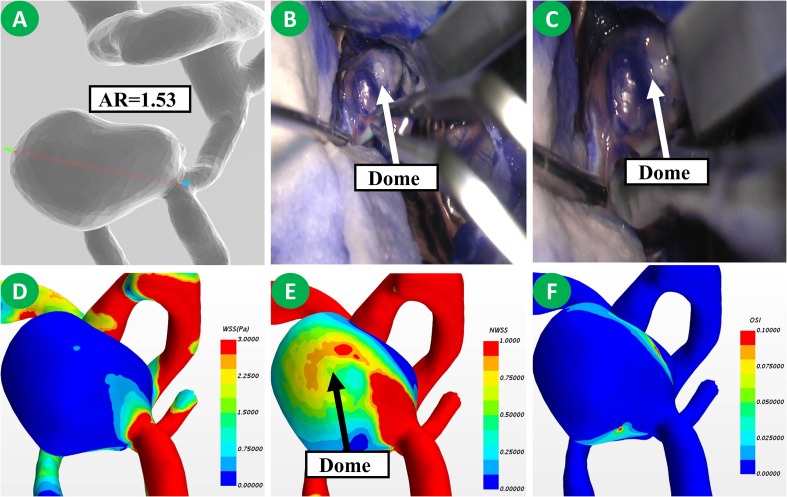
This is a patient with non-IOR aneurysm. Its AR was 1.53 **(A)**. Uneven thickening of the dome of the aneurysm was observed during the operation **(B)**. There existed no rupture during clip application **(C)**. Based on the hemodynamic analysis, the dome of the aneurysm has a low WSS but a higher NWSS **(D,E)**. No significant increase of OSI was observed **(F)**.

The differences in morphological and radiological characteristics were compared. The result revealed that the differences of L (11.5 ± 7.9 vs. 9.7 ± 5.3, *p* = 0.041), D (10.8 ± 6.9 vs. 8.3 ± 4.2, *p* = 0.032), AR (1.8 ± 0.7 vs. 1.2 ± 0.5, *p* < 0.001), and NSI (0.22 ± 0.07 vs. 0.17 ± 0.05, *p* < 0.001) were significant between IOR and non-IOR aneurysms (Table 2). However, no statistically significant difference was found in d (*p* = 0.063), H (*p* = 0.378), SR (*p* = 0.518), UI (*p* = 0.202), EI (*p* = 0.530), multiple aneurysms (*p* = 0.949) and daughter sac (*p* = 0.059), suggesting that the AR of an IOR aneurysm is obviously higher than that of a non-IOR aneurysm (Figure 2A, 3A). The result of morphological and radiological differences is given in Table 2.

**Table 2:** Morphological and radiological characteristics, aneurysms’ hemodynamic characteristics.

	IOR	Non-IOR	*P*
	
Characteristics	*n* = 96	*n* = 96	Value
**Morphological and radiological characteristics**			
L (mm)^∗^	11.5 ± 7.9	9.7 ± 5.3	0.041
D (mm)^∗^	10.8 ± 6.9	8.3 ± 4.2	0.032
d (mm)	7.9 ± 4.6	6.9 ± 3.4	0.063
H (mm)	9.7 ± 6.1	8.4 ± 5.2	0.378
AR^∗^	1.8 ± 0.7	1.2 ± 0.5	<0.001
SR	2.4 ± 1.4	2.2 ± 0.9	0.518
UI	0.10 ± 0.03	0.11 ± 0.04	0.202
EI	0.16 ± 0.06	0.12 ± 0.05	0.530
NSI^∗^	0.22 ± 0.07	0.17 ± 0.05	<0.001
Multiple aneurysms			0.949
YES	14	18	
NO	82	78	
Daughter sac			0.059
YES	19	7	
NO	77	89	
Localization			N/A
Internal carotid artery	11	11	
Middle cerebral artery	27	27	
Anterior cerebral artery	14	14	
Anterior communicating artery	32	32	
Posterior circulation	2	2	
**Aneurysms’ hemodynamic characteristics**			
NPa	0.61 ± 0.22	0.60 ± 0.12	0.543
NPm	0.92 ± 0.32	0.89 ± 0.18	0.501
NWSSa	0.29 ± 0.20	0.31 ± 0.16	0.512
NWSSm^∗^	0.48 ± 0.25	0.95 ± 0.64	<0.001
WSSG (Pa/m)	13.73 ± 7.06	11.94 ± 6.75	0.096
LSAR	0.40 ± 0.23	0.35 ± 0.23	0.121
OSI^∗^	0.044 ± 0.054	0.018 ± 0.005	<0.001
RRT	7.81 ± 4.59	7.69 ± 2.63	0.392

*^∗^Is the parameter with statistically significance; “N/A” is the parameter without further evaluation.*

### Hemodynamic Differences

Comparing the hemodynamic characteristics, statistically significant differences were only found in NWSSm (0.48 ± 0.25 vs. 0.95 ± 0.64, *p* < 0.001) and OSI (0.044 ± 0.054 vs. 0.018 ± 0.005, *p* < 0.001). However, the differences of NPa (*p* = 0.543), NPm (*p* = 0.501), NWSSa (*p* = 0.512), WSSG (*p* = 0.096), LSAR (*p* = 0.121) and RRT (*p* = 0.392) were not statistically significant. The result revealed that the NWSSm is much lower, and the OSI of IOR aneurysms is obviously higher than those of non-IOR aneurysms (Figure 2D–F, 3D–F). The result of hemodynamic differences is listed in Table 2.

**FIGURE 3:**
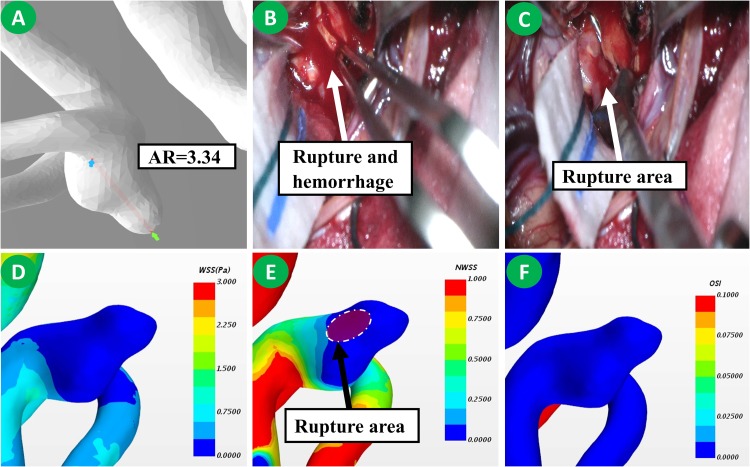
This is a patient with IOR aneurysm. Its AR was 3.34 **(A)**. Rupture of the aneurysm occurs during dissection **(B)**. No significant thinning of aneurysms was observed during the operation, yet the bleeding area could be found **(C)**. Based on the hemodynamic analysis, the dome of the aneurysm has a low WSS but a lower NWSS. The rupture area was in the low WSS region **(D,E)**. No significant increase of OSI was observed **(F)**.

### Result of Univariate and Multivariate Logistic Regression Analysis

Using the significant different variables, a univariate logistic analysis was conducted. The result revealed that AR (OR = 4.20, *p* < 0.001), NSI (OR = 11.35, *p* < 0.001), NWSSm (OR = 11.15, *p* < 0.001) and OSI (OR = 58.21, *p* = 0.001) were risk factors for IOR. By introducing those factors into a multivariable logistic analysis, the result verified that AR (OR = 7.03, *p* < 0.001), NWSSm (OR = 15.55, *p* = 0.014) and OSI (OR = 28.30, *p* < 0.001) are independent risk factors for IOR. The result is summarized in Table 3.

**Table 3:** The result of logistic regression analysis.

Variable	OR	Univariate logistic regression *p*-value	OR	Multivariate logistic regression *p*-value
L	3.80	0.059	**–**	**–**
D	1.07	0.220	**–**	**–**
AR^∗^	4.20	<0.001	7.03	<0.001
NSI	11.35	<0.001	9.33	0.053
NWSSm^∗^	11.15	<0.001	15.55	0.014
OSI^∗^	58.21	0.001	28.30	<0.001

*^∗^Is the independent risk factor.*

### The Morphological and Hemodynamic Differences of Different Rupture Stages

**FIGURE 4:**
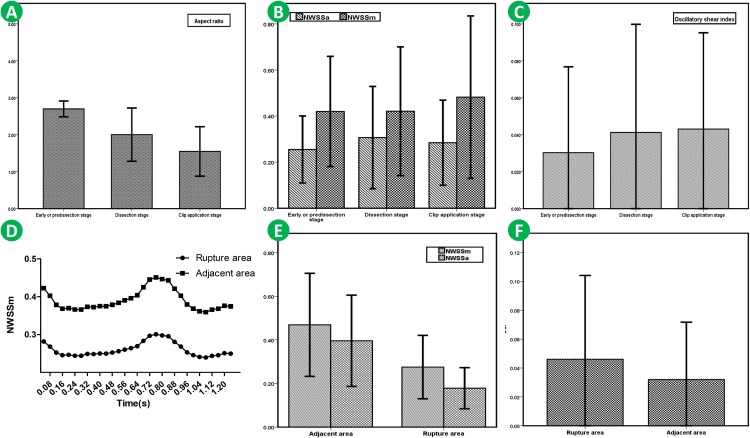
AR for IAs ruptured in early or pre-dissection stage was much larger than that for IAs ruptured in dissection stage or clip application stage **(A)**. According to the trendency of NWSSm and NWSSa, with the decrease in WSS, the risk of early IOR (rupture before clip application) is higher. However, both NWSSa and NWSSm were very similar for IAs ruptured in dissection stage or clip application stage **(B)**. OSI for the IAs ruptured in early or pre-dissection stage seems lower than that for the IAs ruptured in other stages **(C)**. However, the difference is of no significance. NWSSa and NWSSm in rupture area were both lower than those in the adjacent area **(D,E)**. OSI in rupture area appears to be higher than that in adjacent area **(F)**.

The hemodynamic differences of different rupture stages were further compared. A statistically significant difference was found in AR (*p* < 0.001), NWSSa (*p* = 0.042) and NWSSm (*p* = 0.002). The result revealed that the AR for IAs ruptured in the early or pre-dissection stage was much larger than that for IAs that ruptured in the dissection stage or clip application stage (Figure 4A). According to the trend of NWSSm and NWSSa (Figure 4B), the risk of early IOR (rupture before clip application) increases with the obvious decrease in WSS. However, both NWSSa and NWSSm were very similar for IAs that ruptured in the dissection or clip application stage. The OSI was similar for IOR aneurysms at different rupture stages (Figure 4C). The result is listed in Table 4.

**Table 4:** The morphological and hemodynamic differences of different rupture stages, and the hemodynamic characteristics of different region in IOR aneurysm.

Variables	Early or pre-dissection stage		Dissection stage	Clip application stage	*p*-value
**The morphological and hemodynamic differences of different rupture stages**					
**AR**^∗^	**2.7 ± 0.4**		**2.0 ± 0.7**	**1.8 ± 0.8**	**<0.001**
**NWSSa**^∗^	**0.24 ± 0.17**		**0.31 ± 0.14**	**0.29 ± 0.11**	**0.042**
**NWSSm**^∗^	**0.42 ± 0.24**		**0.43 ± 0.28**	**0.49 ± 0.35**	**0.002**
OSI	0.030 ± 0.046		0.042 ± 0.059	0.043 ± 0.052	0.646

**Variables**		**Rupture area**		**Adjacent area**	***p*-value**

**The hemodynamic characteristics of different region in IOR aneurysm**					
**NWSSa**^∗^		**0.18 ± 0.12**		**0.39 ± 0.11**	**<0.001**
**NWSSm**^∗^		**0.26 ± 0.15**		**0.46 ± 0.22**	**<0.001**
OSI		0.048 ± 0.048		0.032 ± 0.039	0.365

*^∗^Is the parameter with statistically significance.*

### The Hemodynamic Characteristics of the Different Regions in IOR Aneurysm

The hemodynamic characteristics of the different regions in an IOR aneurysm were also compared. The result indicated that NWSSa and NWSSm in the rupture area were both lower than those in adjacent areas (Figure 4D,E). The differences were statistically significant (*p* < 0.001). OSI in the rupture area was higher than that in adjacent areas (Figure 4F). However, the difference failed to be of significance (*p* = 0.365). The result is given in Table 4.

## Discussion

IOR is a difficult event during the clipping for IAs and can result in a bad prognosis (Xie et al., 2014; Chen et al., 2016), therefore, preoperative discrimination of aneurysms at a high risk of IOR, is important for decision-making. For these IAs, care should be taken during operation and in the selection of interventions. By analyzing the morphological and hemodynamic characteristics of IOR aneurysms, a morphological parameter and two hemodynamic parameters related to the IOR were found.

Intraoperative rupture has a low incident rate, yet the rate reported in previous studies is quite different. A previous study reported an IOR incident rate of 2.63%, 9.6% for ruptured aneurysm and 0.57% for unruptured aneurysm (Chen et al., 2016). However, this result is far from the 14.7% rate reported in another study (Lakicevic et al., 2015). Several relevant studies in recent years were further summarized, and the incident rate of IOR during clipping was found to vary between 2.63 and 22.2% (Xie et al., 2014; Zhen et al., 2014; Lakicevic et al., 2015; Burkhardt et al., 2016; Hsu et al., 2016; Jun et al., 2016). Except the risk factors, e.g., presurgical rupture history and localization (Schramm and Cedzich, 1993; Leipzig et al., 2005; Lakicevic et al., 2015; Darkwah Oppong et al., 2018), the main reason for this difference is the definition of IOR. Previous authors defined IOR as bleeding that interrupts the microsurgical procedure and excluded the trivial leaks that are easily dealt with by most neurosurgeons, or minor hemorrhages during clip application, which were controlled by simply closing the clip blades (Giannotta et al., 1991; Chandler et al., 1998). However, any unintended leak here indicates a fragile aneurysm wall, all of which should be considered in our present study. The incident rate of IOR in our series was up to 4.3%, which is also quite low.

The irregular morphological characteristic was associated with IOR. The AR, the ratio of length to neck width, could reflect the irregular shape of an aneurysm (Dhar et al., 2008; Duan et al., 2016; Huhtakangas et al., 2017; Juvela and Korja, 2017). An aneurysm with an irregular shape is usually at a high risk of natural rupture (Kleinloog et al., 2017; Goertz et al., 2018). Qiu et al. (2017) found severe pathological features in IAs with larger AR. In a radiological study, a larger AR was found to be a predictor for IOR (Gu et al., 2017). Accordingly, those aneurysms with a larger AR are at a higher risk of both natural rupture and IOR. Our finding suggested a similar experience that an aneurysm with a larger AR has a higher risk of IOR. The AR of IOR aneurysms at different stages was further compared. The result suggested that the AR of aneurysms that ruptured at the early or pre-dissection stage, was much larger than that of aneurysms which ruptured at the dissection and clip application stage. However, the AR of an aneurysm which ruptured at the dissection and clip application stage was similar. For this phenomenon, it is easy to understand that aneurysms that ruptured at the early stages were not subjected to any external force. Thus, the mechanism of early rupture might be similar to a naturally ruptured aneurysm, which is caused by a fragile aneurysm wall. We thought that aneurysms that ruptured at the early or pre-dissection stage were also at a higher risk of natural rupture, but, an aneurysm that ruptured at a late stage, such as the dissection stage and clip application stage, can still withstand external forces. Those aneurysms have a lower AR. Thus, the larger the AR, the greater the risk of aneurysm rupture will be, during clipping.

Hemodynamic characteristics are also risk factors for IOR. Multivariate logistic analysis proved NWSSm and OSI as independent risk factors. NWSSm is the normalized maximum value of the wall shear stress of the systolic phase. In this study, low WSS was found to be related to IOR. Low WSS was considered to trigger the inflammatory-cell-mediated pathway, making the aneurysm large and thick (Meng et al., 2014). Those pathological processes could lead to a destructive remodeling, which results in atherosclerosis and/or thrombus formation in the aneurysm wall. Thus, a long period of low WSS has a destructive effect on the aneurysm wall. This phenomenon was also found in an IOR aneurysm (Yoshiki et al., 2017). In this study, we found that an IOR aneurysm has a much lower NWSSm, consistent with the relationship between low WSS and aneurysm wall degeneration. However, the NWSSa was not significantly different between IOR aneurysms and non-IOR aneurysms. This is because the IAs that we choose to intervene with are the ones that are at higher risk of natural rupture. Low WSS is one of the main hemodynamic characteristics of a naturally ruptured aneurysm (Xiang et al., 2011), suggesting that such aneurysms may rupture in the following period even without an intervention. Here, aneurysms with low NWSSa were found prone to rupture at the early or pre-dissection stage (NWSSa of IOR aneurysms at early or pre-dissection stage was much lower than that of IOR aneurysms at dissection and clip application stage). This fact proves that the lower WSS aneurysm itself has a high risk of rupture. Thus, the NWSSa failed to show a significant difference between IOR aneurysms and non-IOR aneurysms, but showed a significant difference in different rupture stages. On the other hand, higher NWSSm may also indicate that the aneurysm may not reach the terminal stage. WSS decreases the irregular shape of the aneurysm (Xiang et al., 2011; Meng et al., 2012). This study found that the ruptured area has a much lower NWSSa and NWSSm compared with the adjacent areas. Thus, it is reasonable to think that low WSS is associated with aneurysm rupture. NWSSm may also decrease gradually as the injury increases, and when the limit is reached, the aneurysm may rupture intraoperatively. Thus, this parameter is vital to identify the risk of IOR.

The other finding was, OSI, which was used to describe the local stability of hemodynamic condition. As previously discussed in Meng’s study, high OSI could cause endothelial surface adhesion molecules to upregulate and resulted in the dysfunction of flow nitrous oxide, increasing endothelial permeability, and final atherogenesis (Xiang et al., 2011). All those pathological aspects could lead to a natural aneurysm rupture (Arnal et al., 1999; Plank et al., 2006). Our study found that an IOR aneurysm has a higher OSI compared with a non-IOR aneurysm. This suggests that high OSI can cause aneurysm wall degeneration, which is also an important factor in the cause of an IOR.

For aneurysms with a higher risk of IOR, the choice of intervention is particularly important. If the aneurysm rupture occurs during coil embolization, the patient is at a high risk of poor prognosis (Kang et al., 2010; Stapleton et al., 2015). Thus, it is very important to judge the risk of an aneurysm rupture before the operation and to select an appropriate treatment method. When the aneurysm ruptures intraoperatively, microsurgical clippings not only obstruct the aneurysm, but also prevent the occurrence of hemorrhage and hematoma, which cannot be achieved by coil embolization. Based on the result here, we suggest that surgical clipping could be the best choice for aneurysms with a high AR, low NWSSm, and high OSI.

The prognosis and appropriate intervention methods have always been the focus of attention in the treatment of IAs. By CFD analysis here, the result could help neurosurgeons select reasonable and effective treatment methods and in further studies, this method can be used to build a simple and effective model for clinical decision-making.

## Conclusion

AR, NWSSm, and OSI were independent risk factors for intraoperative aneurysm rupture, which could be used as predictors. A selection of intervention methods for aneurysms with high AR, low NWSSm, and high OSI should be carefully considered. For IAs with high AR, low NWSSm, and high OSI, surgical clipping is the best choice.

## Limitations

There were several limitations in this study. First, this is a retrospective study and the conclusion here was limited and requires further studies in order to provide more evidence. Second, some risk factors were balanced here using the PSM based on previous studies. However, potential risk factors affecting the hemodynamic condition in aneurysms may exist, making selection bias hard to avoid. This study attempted to explain the role of hemodynamic and morphological features in IOR. It is believed that this conclusion is close to the real condition of IOR aneurysms. Third, all morphological parameters were calculated from 3-dimensional CTAs, which can exclude the effects of thrombus, but do not provide the essential image like 3-dimensional angiography. Additionally, all hemodynamic models were reconstructed from a 3-dimensional CTA, which may affect the accuracy of our simulation. Collaboration with the angiogram can improve results, yet the angiogram was not routinely performed in the clinical work. Thus, this work represents our best efforts. Fourth, some clinical characteristics were not considered, e.g., multi-IAs and IAs with vascular stenosis. For patients with multi-IAs, we did not explore the features of the IOR aneurysm compared to a non-IOR aneurysm. However, considering that this study mainly investigated the differences between IOR and non-IOR aneurysms, further studies are required to explore these features.

The CFD analysis here also had several limitations. The inlet boundary condition was from a representative patient, which could affect the result of CFD since this method is sensitive to velocity and waveform (Xiang et al., 2014). However, this study tried to use normalized parameters to reduce the effects of this problem (Xiang et al., 2014). In a future study, a reliable ultrasonic expert may be recruited, and all questions discussed may be solved. However, multiple research groups are trying to use CFD analysis to help neurosurgeons select reasonable and effective treatment methods. It is believed that the CFD analysis here may help physicians with decision-making in the future.

## Ethics Statement

The study was approved by the institutional review board of the Beijing Tiantan hospital (Ethical No. KY2017-076-01). Written informed consent was obtained from all participants or their legally authorized representatives and the privacy of patients was effectively protected.

## Author Contributions

QL and PJ were responsible for data collection, and drafting and revising the manuscript. JW provided the statistical support. BG provided the technical support. SW supervised the study.

## Conflict of Interest Statement

The authors declare that the research was conducted in the absence of any commercial or financial relationships that could be construed as a potential conflict of interest.

## Abbreviations

AR: aspect ratio
d: diameter of neck
D: maximum diameter of body
EI: ellipticity index
H: perpendicular height
L: maximum length
NPa: normalized pressure average
NPm: normalized pressure maximum
NSI: non-sphericity index
NWSSa: normalized WSS average
NWSSm: normalized WSS maximum
OSI: oscillatory shear index
RRT: relative resident time
SR: size ratio
UI: undulation index
WSS: wall shear stress.

## References

[B1] Arnal F. Dinh-Xuan A. T. Pueyo M. Darblade B. Rami J. (1999). Endothelium-derived nitric oxide and vascular physiology and pathology. *Cell Mol. Life Sci.* 55 1078–1087. 10.1007/s000180050358 10442089 PMC11146781

[B2] Batjer H. Samson D. (1986). Intraoperative aneurysmal rupture: incidence, outcome, and suggestions for surgical management. *Neurosurgery* 18 701–707. 10.1227/00006123-198606000-00004 3736796

[B3] Burkhardt J. K. Neidert M. C. Mohme M. Seifert B. Regli L. Bozinov O. (2016). Initial clinical status and spot sign are associated with intraoperative aneurysm rupture in patients undergoing surgical clipping for aneurysmal subarachnoid hemorrhage. *J. Neurol. Surg. A Cent. Eur. Neurosurg.* 77 130–138. 10.1055/s-0035-1558414 26216733

[B4] Chandler J. P. Getch C. C. Batjer H. H. (1998). Intraoperative aneurysm rupture and complication avoidance. *Neurosurg. Clin. N. Am.* 9 861–868. 10.1016/S1042-3680(18)30234-1 9738112

[B5] Chen S. F. Kato Y. Kumar A. Tan G. W. Oguri D. Oda J. (2016). Intraoperative rupture in the surgical treatment of patients with intracranial aneurysms. *J. Clin. Neurosci.* 34 63–69. 10.1016/j.jocn.2016.01.045 27692502

[B6] Darkwah Oppong M. Pierscianek D. Ahmadipour Y. Dinger T. F. Dammann P. Wrede K. H. (2018). Intraoperative aneurysm rupture during microsurgical clipping: risk re-evaluation in the post-international subarachnoid aneurysm trial era. *World Neurosurg.* 119 e349–e356. 10.1016/j.wneu.2018.07.158 30059784

[B7] Dhar S. Tremmel M. Mocco J. Kim M. Yamamoto J. Siddiqui A. H. (2008). Morphology parameters for intracranial aneurysm rupture risk assessment. *Neurosurgery* 63 185–96; discussion196–197. 10.1227/01.NEU.0000316847.64140.81 18797347 PMC2570753

[B8] Duan G. Lv N. Yin J. Xu J. Hong B. Xu Y. (2016). Morphological and hemodynamic analysis of posterior communicating artery aneurysms prone to rupture: a matched case-control study. *J. Neurointerv. Surg.* 8 47–51. 10.1136/neurintsurg-2014-011450 25404406

[B9] Farnoush A. Avolio A. Qian Y. (2013). Effect of bifurcation angle configuration and ratio of daughter diameters on hemodynamics of bifurcation aneurysms. *AJNR Am. J. Neuroradiol.* 34 391–396. 10.3174/ajnr.A3222 22859285 PMC7965116

[B10] Giannotta S. L. Oppenheimer J. H. Levy M. L. Zelman V. (1991). Management of intraoperative rupture of aneurysm without hypotension. *Neurosurgery* 28 531–535. 10.1227/00006123-199104000-00008 2034347

[B11] Goertz L. Hamisch C. Telentschak S. Kabbasch C. von Spreckelsen N. Stavrinou P. (2018). Impact of aneurysm shape on intraoperative rupture during clipping of ruptured intracranial aneurysms. *World Neurosurg.* 118 e806–e812. 10.1016/j.wneu.2018.07.058 30031199

[B12] Gu Y. Xu L. Hu C. Luo M. Zhang H. Liu X. (2017). Monitoring dynamic morphological changes with electrocardiography-gated dynamic 4-dimensional computed tomography angiography to predict intraoperative rupture of intracranial aneurysms. *J. Comput. Assist. Tomogr.* 42 286–292. 10.1097/RCT.0000000000000671 28937485

[B13] Hsu C. E. Lin T. K. Lee M. H. Lee S. T. Chang C. N. Lin C. L. (2016). The impact of surgical experience on major intraoperative aneurysm rupture and their consequences on outcome: a multivariate analysis of 538 microsurgical clipping cases. *PLoS One* 11:e0151805. 10.1371/journal.pone.0151805 27003926 PMC4803230

[B14] Huhtakangas J. Lehecka M. Lehto H. Jahromi B. R. Niemela M. Kivisaari R. (2017). CTA analysis and assessment of morphological factors related to rupture in 413 posterior communicating artery aneurysms. *Acta Neurochir.* 159 1643–1652. 10.1007/s00701-017-3263-4 28710522

[B15] Jun L. I. Xiao W. Zhai A. Neurosurgery D. O. Nohospital T. (2016). Prognosis and risk factor analysis of intraoperative aneurysm rupture patients with intracranial aneurysm clipping. *Med. J. Nat. Defend. Forces Northwest China* 38 516–519.

[B16] Juvela S. Korja M. (2017). Intracranial aneurysm parameters for predicting a future subarachnoid hemorrhage: a long-term follow-up study. *Neurosurgery* 81 432–440. 10.1093/neuros/nyw049 28327974

[B17] Kang H. Kim Y. S. Baik S. K. Park S. H. Park J. Hamm I. S. (2010). Acute serious rebleeding after angiographically successful coil embolization of ruptured cerebral aneurysms. *Acta Neurochir* 152 771–781. 10.1007/s00701-009-0593-x 20099070

[B18] Kleinloog R. de Mul N. Verweij B. H. Post J. A. Rinkel G. J. E. Ruigrok Y. M. (2017). Risk factors for intracranial aneurysm rupture: a systematic review. *Neurosurgery* 82 431–440. 10.1093/neuros/nyx238 28498930

[B19] Lakicevic N. Vujotic L. Radulovic D. Cvrkota I. Samardzic M. (2015). Factors influencing intraoperative rupture of intracranial aneurysms. *Turk Neurosurg.* 25 858–885. 10.5137/1019-5149.JTN.12966-14.2 26617133

[B20] Lawton M. T. Vates G. E. (2017). Subarachnoid Hemorrhage. *N. Engl. J. Med.* 377 257–266. 10.1056/NEJMcp1605827 28723321

[B21] Leipzig T. J. Morgan J. Horner T. G. Payner T. Redelman K. Johnson C. S. (2005). Analysis of intraoperative rupture in the surgical treatment of 1694 saccular aneurysms. *Neurosurgery* 56 455–468. 10.1227/01.NEU.0000154697.75300.C2 15730570

[B22] Meng H. Tutino V. M. Xiang J. Siddiqui A. (2014). High WSS or low WSS? complex interactions of hemodynamics with intracranial aneurysm initiation, growth, and rupture: toward a unifying hypothesis. *AJNR Am. J. Neuroradiol.* 35 1254–1262. 10.3174/ajnr.A3558 23598838 PMC7966576

[B23] Meng H. Xiang J. Liaw N. (2012). The role of hemodynamics in intracranial aneurysm initiation. *Int. Rev. Thromb.* 7 40–57.

[B24] Plank J. Wall D. J. David T. (2006). Atherosclerosis and calcium signalling in endothelial cells. *Prog. Biophys. Mol. Biol.* 91 287–313. 10.1016/j.pbiomolbio.2005.07.005 16171849

[B25] Qiu T. Jin G. Xing H. Lu H. (2017). Association between hemodynamics, morphology, and rupture risk of intracranial aneurysms: a computational fluid modeling study. *Neurol. Sci.* 38 1009–1018. 10.1007/s10072-017-2904-y 28285454 PMC5486504

[B26] Schramm J. Cedzich C. (1993). Outcome and management of intraoperative aneurysm rupture. *Surg. Neurol.* 40 26–30. 10.1016/0090-3019(93)90165-W 8322173

[B27] Stapleton J. Walcott B. P. Butler W. E. Ogilvy C. S. (2015). Neurological outcomes following intraprocedural rerupture during coil embolization of ruptured intracranial aneurysms. *J. Neurosurg.* 122 128–135. 10.3171/2014.9.JNS14616 25361491

[B28] Tian Z. Zhang Y. Jing L. Liu J. Zhang Y. Yang X. (2016). Rupture risk assessment for mirror aneurysms with different outcomes in the same patient. *Front. Neurol.* 7:219. 10.3389/fneur.2016.00219 27994571 PMC5136536

[B29] Walendy V. Stang A. (2017). Clinical management of unruptured intracranial aneurysm in germany: a nationwide observational study over a 5-year period (2005–2009). *BMJ Open* 7:e012294. 10.1136/bmjopen-2016-012294 28096250 PMC5253577

[B30] Xiang J. Natarajan S. K. Tremmel M. Ma D. Mocco J. Hopkins L. N. (2011). Hemodynamic-morphologic discriminants for intracranial aneurysm rupture. *Stroke* 42 144–152. 10.1161/STROKEAHA.110.592923 21106956 PMC3021316

[B31] Xiang J. Siddiqui A. H. Meng H. (2014). The effect of inlet waveforms on computational hemodynamics of patient-specific intracranial aneurysms. *J. Biomech.* 47 3882–3890. 10.1016/j.jbiomech.2014.09.034 25446264 PMC4261154

[B32] Xie W. F. Wang J. Chuan-Kun L. I. Neurosurgery D. O. (2014). Ananalysis of risk factors and response measures of intraoperative intracranial aneurysm rupture. *J. Clin. Neurosurg.* 11 181–183.

[B33] Yoshiki K. Misaki K. Nambu I. Fukui I. Mohri M. Uchiyama N. (2017). Intraoperative rupture of unruptured cerebral aneurysm during craniotomy: a case report. *Case Rep. Neurol.* 9 261–266. 10.1159/000480425 29422847 PMC5803730

[B34] Zhen Y. Yan K. Zhang H. Zhao S. Xu Y. Zhang H. (2014). Analysis of the relationship between different bleeding positions on intraoperative rupture anterior circulation aneurysm and surgical treatment outcome. *Acta Neurochir.* 156 481–491. 10.1007/s00701-013-1953-0 24322582

